# Characterizing #Backwoods on Instagram: “The Number One Selling All Natural Cigar”

**DOI:** 10.3390/ijerph17124584

**Published:** 2020-06-25

**Authors:** Sabrina L. Smiley, Stephanie Kim, Alia Mourali, Jon-Patrick Allem, Jennifer B. Unger, Tess Boley Cruz

**Affiliations:** Department of Preventive Medicine, Keck School of Medicine, University of Southern California, Los Angeles, CA 90032-3628, USA; kim587@usc.edu (S.K.); mourali@usc.edu (A.M.); allem@usc.edu (J.-P.A.); unger@usc.edu (J.B.U.); tesscruz@usc.edu (T.B.C.)

**Keywords:** cigar, Instagram, natural, misleading

## Abstract

We sought to assess the proportion of Backwoods (Imperial Tobacco Group Brands LLC) cigar-related posts to Instagram that may contain misleading claims, nature-evoking imagery, and appealing flavors. Inclusion criteria for this study included an Instagram post with the hashtag “#backwoods” from 30 August to 12 September 2018. Rules were established to content analyze (n = 1206) posts. Categories included *misleading packaging* (i.e., the post contained an image of a Backwoods product with the descriptor “natural” on the packaging), *misleading promo* (i.e., the corresponding caption to the post contained hashtag(s) like “#natural”, “#authentic”, “#alwaystrue”), *nature*-*evoking imagery* (i.e., the post contained images of grass, water, and pastural views along with a Backwoods product), *flavors* (i.e., the post contained a Backwoods product with brand-specific flavors on the packaging), *flavor promo* (i.e., the corresponding caption to the post contained hashtag(s) of Backwoods’ brand-specific flavors), *marijuana*-*related* (i.e., the post contained an image of marijuana next to a Backwoods pack, rolled cigars visibly contained marijuana, or hollowed-out cigars next to marijuana), *smoking* (the post contained an image of smoke or a lit cigar), *brand*-*specific promo* (i.e., the post contained an image of a Backwoods t-shirt, sweatshirt, hat, etc.), and perceived gender. Among the posts analyzed, 645 (53.5%) were *marijuana*-*related*, 564 (46.8%) were *flavors*, 463 (38.4%) were *misleading packaging*, 335 (27.8%) were *flavor promo*, 309 (25.6%) were *misleading promo*, 188 (15.6%) were *nature*-*evoking* imagery, 165 (13.7%) were *smoking*, 157 (13.0%) were *brand*-*specific promo*, and 239 (19.8%) were *perceived male gender*. Backwoods cigar-related posts to Instagram often contained misleading images and promotions of a “natural” tobacco product, images of marijuana use (in the form of blunt-making), brand-specific flavors, smoking, and promotional merchandise. Misleading images and the depictions of marijuana use in addition to the variety of flavor options may increase product appeal to consumers. These results underscore the need for comprehensive regulation of cigar products similar to cigarettes.

## 1. Introduction

Trends in cigarette sales have declined in the U.S. in recent years; however, sales of machine-manufactured, mass-merchandised little cigars and cigarillos have been on the rise, especially since flavored cigarettes were banned (with the exception of menthol) as part of the 2009 Family Smoking Prevention and Tobacco Control Act [1,2]. Little cigars and cigarillos comprise more than 90% of the cigar market, whereas traditional premium cigars constitute less than 10% [1]. Less stringent sales and marketing regulations may explain the large market share in little cigar and cigarillo sales [1]. Little cigars and cigarillos are less expensive than cigarettes, available in a variety of appealing flavors, and have no minimum pack size standards [1,3]. In other words, consumers are able to buy a single honey-flavored cigarillo for less than $1.00, whereas cigarettes are sold in the U.S. in packages of 20 and at higher prices [1,3].

Disparities exist in little cigar and cigarillo use patterns. For example, current (past 30 days) little cigar and cigarillo smokers are more likely to identify as male gender, racial or ethnic minority, use marijuana, and smoke cigarettes [4,5,6,7], thus, exposing themselves to considerable amounts of nicotine and other harmful components of tobacco smoke [8,9,10]. This could mean greater potential for increased nicotine dependence, decreased quitting success, and an increased risk of tobacco-related cancers.

**Figure 1:**
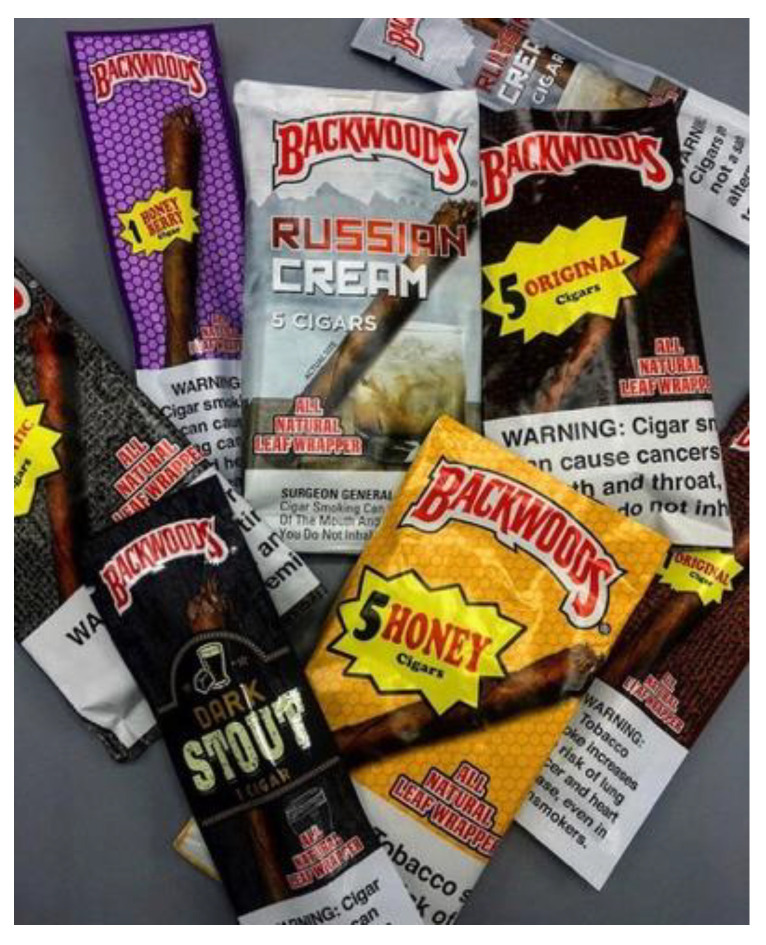
Backwoods cigar packaging.

Backwoods (Figure 1), a machine-manufactured, mass-merchandised cigar brand (Imperial Tobacco Group [ITG] Brands LLC–the third largest tobacco company in the U.S.) with a rising market share. Backwoods uses words and phrases like “natural” and “always true” in its advertising claims and product packaging [11]. Backwoods’ brand-specific flavors (e.g., black n’ sweet aromatic, dark stout, Russian creme) may appeal to consumers while claims about “natural cigars” on the company’s website [11] may mislead consumers into perceiving that Backwoods offers a reduced risk tobacco product.

The Family Smoking Prevention and Tobacco Control Act granted the Food and Drug Administration (FDA) authority to prohibit all tobacco companies from using marketing, advertising, or promotional claims that suggest reduced exposure to harmful substances [12]. Recently, Santa Fe Tobacco Company (Reynolds American Incorporated) responded to the FDA concerns by eliminating the use of the terms “additive-free” and “natural” to describe American Spirit cigarettes in advertising and on packaging [13]. Similarly, ITG Brands is potentially deceiving consumers with Backwoods’ advertising claims (i.e., “always true”) and packaging descriptors (i.e., “all natural leaf wrapper”) that suggests their cigars are less risky than other cigars or cigarettes. This is especially concerning since studies have shown that consumers perceive cigars to be more “natural” and less harmful than cigarettes [2,14,15,16].

In the current study, we examined Backwoods cigar-related posts to Instagram. Instagram offers a unique opportunity to see how tobacco users engage with tobacco products, including cigars. Technological advances have changed the way in which companies communicate with consumers. Instagram, an image-based social media app with over one billion active (monthly) users [17], allows users (including companies) to capture, customize, and post photos on the Internet. Instagram is the second ranked social media app among active users, behind Facebook [17], and plays an important role in consumer behavior [18]. Given the lack of data on cigar marketing practices on social media [19], in particular brand-specific data, findings from this study can inform tobacco control and prevention by providing insights into the types of cigar-related messages that viewers are exposed to on the Internet.

## 2. Materials and Methods

All data were collected through Netlytic, an Instagram-approved third-party vendor. Netlytic uses an auto-collector that retrieves 100 posts every hour (around 30 min past the hour). If there were more than 100 posts with a particular hashtag posted per hour, only the most recent would be retrieved until capacity is reached. Netlytic has been used in prior research focused on tobacco companies’ communications on Instagram [20]. All data collected were publicly available; that is, anyone with an Internet connection could have viewed the post at the time it was collected. Only Instagram posts with the hashtag #backwoods were included. It is common for people to refer to little cigars and cigarillos by their brand name (e.g., Backwoods, Swisher, White Owl) on social media [19,21], and to our knowledge, no study has focused on Backwoods “natural” cigar promotion, justifying this inclusion criterion and the need to examine this specific brand. Data collection occurred from 30 August 2018 to 12 September 2018. A total of (n = 12,306) posts included the hashtag #backwoods during the study period. Similar to prior Instagram studies [19,22], a random subsample of posts was then drawn constituting approximately 10% of the original sample. Rules were established to content analyze (n = 1206) posts.

## 3. Coding Strategy

The first and second authors generated a codebook based on prior research [19,23,24] and reviewed a subsample of the posts to identify additional prominent themes. The unit of analysis was an individual Instagram post (i.e., the image and corresponding caption) and the coding strategy assessed characteristics and themes. Categories included *type of post* (i.e., photo or video or both) and *perceived gender* (i.e., individuals in the image were considered to be male or female or unable to tell). Perceived race/ethnicity was considered for coding but excluded because we believed it to be more subject to implicit bias, especially when considered against other variables that were coded, like gender. Other categories included: (1) *misleading packaging* (i.e., the post contained an image of a Backwoods product with the descriptor “natural” on the packaging; (2) *misleading promo* (i.e., the corresponding caption to the post contained hashtag(s) like “#natural”, “#authentic”, “#alwaystrue”); (3) *nature*-*evoking imagery* (i.e., the post contained images of grass, water, and pastural views along with a Backwoods product); (4) *flavors* (i.e., the post contained an image of a Backwoods product with brand-specific flavors like honey, sweet aromatic, and dark stout on the packaging); (5) *flavor promo* (i.e., the corresponding caption to the post contained hashtag(s) of Backwoods’ brand-specific flavors); (6) *marijuana*-*related* (i.e., the post contained an image of marijuana next to a Backwoods pack, rolled cigars visibly contained marijuana, or hollowed-out cigars next to marijuana); (7) *smoking* (i.e., the post contained an image of an individual(s) blowing smoke or a lit Backwoods cigar); and (8) *brand*-*specific promo* (the post contained an image of a Backwoods t-shirt, sweatshirt, hat, etc.). The second and third authors independently coded all posts and the first author double-coded a random subsample of posts (n = 200) to determine reliability. Percentage agreement across all codes was substantial (98% agreement; κ = 0.86) and discrepancies were resolved via in-person discussion. We reported the percentages for each characteristic and theme.

## 4. Results

Among the 1206 posts, 913 (75.7%) were photos, 268 (22.2%) were videos, and 25 (2.1%) included both photos and videos. Perceived gender was coded as unable to tell (n = 279 or 23.1%), followed by male (n = 239 or 19.8%), and female (n = 111 or 9.2%), while other posts did not contain images of people (n = 520 or 44.8%).

**Figure 2:**
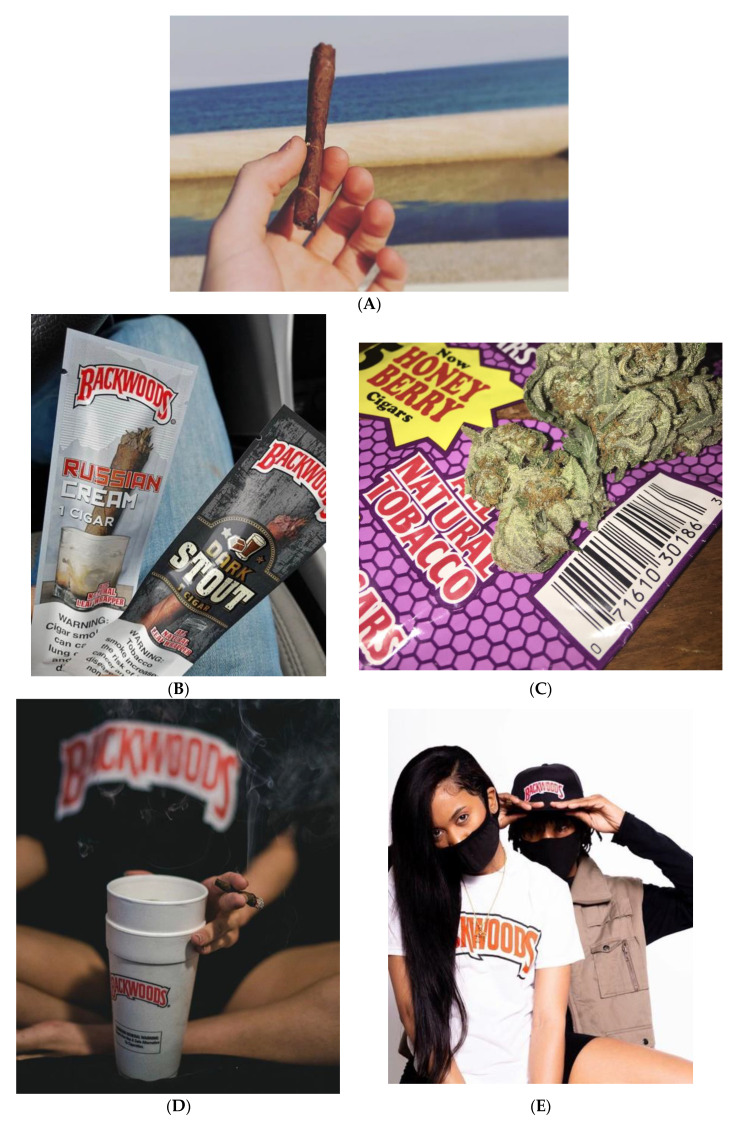
Images representative of select themes: (**A**) nature-evoking imagery; (**B**) brand-specific flavors; (**C**) marijuana-related; (**D**) smoking; (**E**) brand-specific promotion.

Among all posts, 463 (38.4%) were coded *misleading packaging*, 309 (25.6%) were *misleading promo*, and 188 (15.6%) were *nature*-*evoking imagery* (Figure 2A). Among all of the posts, 564 images (46.8%) were coded *flavors* (Figure 2B), and 335 (27.8%) were coded *flavor promo* (e.g., dark stout, honey, sweet aromatic). Among all images, 645 (53.5%) were *marijuana*-*related* (Figure 2C), 165 (13.7%) were *smoking* (Figure 2D), and 157 (13.0%) were coded as *brand*-*specific promo* (Figure 2E).

## 5. Discussion

Backwoods cigar-related posts to Instagram often contained imagery suggestive of a “natural” tobacco product as well as posts that highlighted flavors, smoking, and promotional merchandise. More than half of posts showed Backwoods cigars paired with marijuana in the form of blunts. Considering that Instagram is a widely used social media platform, posts described in this study may increase brand awareness and normalize cigar-related attitudes and behaviors.

Positive promotion of Backwoods on Instagram, including nature-related images and hashtags, may have an impact on consumers’ normative expectations and subsequent behaviors [25]. Exposure to images, descriptors, and advertising claims on Instagram could make Backwoods’ products appealing to non-smokers. Pack descriptors such as “all natural tobacco leaf” and “always true” may provide consumers with a false sense of reduced risk as well as discourage quit attempts among smokers. Emerging scientific evidence [26,27,28] has demonstrated that cigarette packages containing the descriptors, “natural,” “organic,” and “additive-free” can mislead consumers. The 2009 Tobacco Control Act gave the FDA authority to regulate false and misleading statements in tobacco advertisements [12]. Our findings suggest that an area of policy consideration for the brand Backwoods would be to restrict the use of claims like “always true” in advertising and “all natural leaf wrapper” on packaging.

Brand-specific flavors (e.g., honey berry, sweet aromatic, dark stout) highlighted on packaging as well as mentioned in hashtags (e.g., #honeyberry, #sweetaromatic, #dark stout) were common among posts. Such findings suggest that there is a need to further regulate cigar flavors to potentially reduce their appeal. Posts promoting sweet flavorings that alter cigar’s sensory effects, may explain the misperceptions of cigars as less harmful relative to cigarettes [2,14,15,16]. Flavorings mask the harsh taste of tobacco which makes it easier for young and novice smokers to initiate tobacco use [29]. Further, the amount of posts in this study containing brand-specific flavor packaging and hashtags may indicate that individuals may promote flavors to their followers on Instagram, demonstrating the appeal of Backwoods cigars. However, further investigation is warranted, for example, empirical data are needed to determine whether a “natural” descriptor coupled with a flavored Backwoods cigar image or message impacts perceived harm, product appeal, intention to use, and actual use.

Our study shows that Backwoods are often used to administer marijuana among Instagram users, which is similar to findings from an earlier study focused on Swisher-related posts to Instagram [19]. It is notable that Backwoods “all natural” tobacco-leaf wrappers are designed to facilitate blunt-making [30], a process whereby some or all of the tobacco is removed from a cigar then replaced with marijuana and smoked [30]. Future studies identifying how tobacco companies design their products and market them for blunt-making (e.g., perforations in the tobacco-leaf wrappers, colorful, resealable, airtight packaging to hide the smell), are essential to inform comprehensive cigar regulations, interventions, education campaigns, and to ultimately reduce tobacco-related health disparities.

Findings from this study show that Backwoods-specific promotional items, including t-shirts, sweatshirts, and hats help establish the brand. This is concerning given that exposure to tobacco promotional items has been associated with smoking initiation and maintenance among youth [31]. The 1998 Master Settlement Agreement and Smokeless Master Settlement Agreement [32] and the 2009 Family Smoking Prevention and Tobacco Control Act [33] included similar restrictions on the use of brand name, logo, symbols, etc., in cigarette and smokeless tobacco items and services; however, this does not include cigars. These restrictions should be extended to cigar products, especially since the final deeming regulation [34] in 2016 extended the FDA’s regulatory authority to all tobacco products, including cigars.

Future studies are needed to understand consumers’ perceptions of Backwoods promotions and products, smoking portrayals on Instagram, and motivations for smoking portrayals by race/ethnicity. Research indicates African Americans are more likely to use little cigars and cigarillos than their racial/ethnic counterparts [4], and past studies demonstrate African American neighborhoods have more marketing for little cigars/cigarillos in the retail environment than other neighborhoods [3,35,36]. Research is also needed to understand cigarillo images on Instagram related to dual use of cigarettes by sociodemographic variables, which was not possible in this study.

## 6. Limitations and Future Research

The present study focused on images from Instagram and findings may not generalize to other social media platforms (i.e., Twitter, Facebook, Tumblr). The images analyzed in this study were collected from a two-week time period and may not generalize to other time periods. Future research should examine a longer timeframe to fully characterize Backwoods-related posts to Instagram. While the focus on one cigar brand limits generalizability of the findings from the present study, such decisions are consistent with prior research studying cigar-related posts to Instagram [19].

## 7. Conclusions

Given that cigars are not regulated with the same restrictions as cigarettes, understanding how consumers use social media to discuss and disseminate potentially misleading information about Backwoods cigars is critical for tobacco control policy and prevention. Backwoods cigar-related images on Instagram serve to expose consumers to brand-specific flavors, promotions (e.g., t-shirts, hats), and pro-smoking messages as well as project cues to smoke. We found that more than half of the images were related to blunt use. Additionally, Instagram posts appear to convey that the Backwoods brand offers a “natural” flavored tobacco product, which may mislead consumers. Misleading promotional practices on Instagram in particular, could pose serious problems for tobacco control, making further empirical research on social media exposure critical.
